# Case Report: Middle lobe syndrome: a rare presentation in eosinophilic granulomatosis with polyangiitis

**DOI:** 10.3389/fimmu.2023.1222431

**Published:** 2023-08-10

**Authors:** Beatrice Maranini, Ippolito Guzzinati, Gian Luca Casoni, Maria Ballotta, Andrea Lo Monaco, Marcello Govoni

**Affiliations:** ^1^ Division of Rheumatology, Department of Medical Sciences, University of Ferrara, Ferrara, Italy; ^2^ Pneumology Unit, Hospital of Rovigo, Rovigo, Italy; ^3^ Section of Anatomic Pathology, Azienda Ospedaliera Rovigo, Rovigo, Italy

**Keywords:** eosinophilic granulomatosis with polyangiitis, EGPA, Churg–Strauss, asthma, middle lobe syndrome

## Abstract

**Background:**

Antineutrophil cytoplasmic antibody (ANCA)-associated vasculitis (AAV) is a group of disorders characterized by necrotizing inflammation of small- and medium-sized blood vessels and the presence of circulating ANCA. Eosinophilic granulomatosis with polyangiitis (EGPA) is a systemic ANCA-associated vasculitis, characterized by peripheral eosinophilia, neuropathy, palpable purpuras or petechiae, renal and cardiac involvement, sinusitis, asthma, and transient pulmonary infiltrates. Middle lobe syndrome (MLS) is defined as recurrent or chronic atelectasis of the right middle lobe of the lung, and it is a potential complication of asthma.

**Case presentation:**

Herein, we describe a case of MLS in a 51-year-old woman, never-smoker, affected by EGPA, presenting exclusively with leukocytosis and elevated concentrations of acute-phase proteins, without any respiratory symptom, cough, or hemoptysis. Chest computed tomography (CT) imaging documented complete atelectasis of the middle lobe, together with complete obstruction of lobar bronchial branch origin. Fiberoptic bronchoscopy (FOB) revealed complete stenosis of the middle lobar bronchus origin, thus confirming the diagnosis of MLS, along with distal left main bronchus stenosis. Bronchoalveolar lavage (BAL) did not detect any infection. Bronchial biopsies included plasma cells, neutrophil infiltrates, only isolated eosinophils, and no granulomas, providing the hypothesis of vasculitic acute involvement less likely. First-line agents directed towards optimizing pulmonary function (mucolytics, bronchodilators, and antibiotic course) were therefore employed. However, the patient did not respond to conservative treatment; hence, endoscopic management of airway obstruction was performed, with chest CT documenting resolution of middle lobe atelectasis.

**Conclusion:**

To the best of our knowledge, this is the first detailed description of MLS in EGPA completely resolved through FOB. Identification of MLS in EGPA appears essential as prognosis, longitudinal management, and treatment options may differ from other pulmonary involvement in AAV patients.

## Introduction

Antineutrophil cytoplasmic antibody (ANCA)-associated vasculitides (AAV) are composite disorders characterized by necrotizing inflammation, predominantly involving small blood vessels, usually associated with circulating myeloperoxidase (MPO)- or proteinase 3 (PR3)-ANCA (1). Medium-sized arteries may also be affected (1).

AAV include granulomatosis with polyangiitis (GPA, formerly Wegener granulomatosis), microscopic polyangiitis (MPA), and eosinophilic granulomatosis with polyangiitis (EGPA, formerly Churg–Strauss syndrome) (1). Taken together, the most commonly affected systems in AAV are the upper airways, lungs, kidneys, eyes, skin, and peripheral nerves. Presenting symptoms include chronic sinusitis, nasal discharge or crusting, hearing loss, ear fullness, tinnitus, cough, wheeze or hemoptysis, renal failure and proteinuria, purpuric rash, and peripheral neuropathy (2). Pulmonary involvement includes necrotizing granulomatous inflammation (nodules, masses, with or without cavitation), tracheobronchial inflammation, alveolar hemorrhage, interstitial lung disease (ILD), and asthma (3).

EGPA is histologically defined by eosinophil-rich, necrotizing granulomatous inflammation, primarily involving the respiratory tract, along with necrotizing vasculitis of small to medium size (4). ANCAs are detected in ~40%–60% of patients with EGPA typically directed against MPO (5).

Middle lobe syndrome (MLS) is a rare clinical entity, defined as chronic or recurrent atelectasis of the right middle lobe of the lung, although it can also involve the lingula of the left lung (6). First described in 1948 (7), it can present in patients of any age. The right middle lobe bronchus is susceptible to partial or total obstruction due to a smaller intraluminal diameter compared to other lobar bronchi (6).

Female patients present smaller intraluminal diameters compared to male counterparts, thus providing anatomical explanation for a female epidemiological predisposition (8).

Here, we report the case of a 51-year-old woman affected by EGPA, presenting exclusively with leukocytosis and elevated concentrations of acute-phase proteins, which turned out to present MLS diagnosis.

## Case presentation

A 51-year-old woman, with an established diagnosis of EGPA, presented to our Rheumatology Clinic for a routine follow-up scheduled visit.

Diagnosis was determined in 2014, when she presented with asthma-like tracheobronchitis, for which she underwent high-resolution chest computed tomography (HRCT) revealing bronchial wall thickening and lung consolidations. MPO-ANCA positivity was evidenced. After pneumological examination, she was referred to bronchoscopy. Bronchoalveolar lavage (BAL) cell count showed 22% eosinophils, while peripheral eosinophilia was >10% on differential white blood cell count. Since IgG4-related disease (IgG4-RD) may cause lung manifestations in terms of interstitial pneumonitis, organizing pneumonia, and lymphomatoid granulomatosis, IgG4 was collected in both serum and tissues, and found within normal ranges. Bronchial mucosa histological samples described granulation tissue, neutrophilic and eosinophilic inflammatory infiltration, plurinucleate giant cells, and fibrinoid necrosis. No peripheral neuropathy or renal involvement was observed at the time. No ear–nose–throat manifestations were referred by the patient; however, and ENT specialist still evaluated her, excluding the presence of rhinological, otological, or other manifestations of EGPA. Finally, diagnosis was established following the 1990 ACR diagnostic criteria for EGPA. The patient’s past medical history highlighted arterial hypertension, thyroid nodules with normal thyroid function, diverticulosis of the sigma, polycystic ovary syndrome (PCOS), and incomplete right bundle branch block. She had never smoked. At disease onset, she was treated with high doses of steroid (prednisone 1 mg/kg/day), gradually tapered until suspension after 6 months. During steroid-tapering, at week 12 from disease onset, therapy with azathioprine was introduced at the dosage of 150 mg and 100 mg every other day (because of moderate toxic myelosuppression at higher doses). The patient remained in remission for 8 years, with a Birmingham Vasculitis Activity Score (BVAS) of 0.

During follow-up visits, neither systemic nor localized symptoms emerged, particularly of the respiratory tract. Her asthma control was optimal with beclometasone-formoterol as maintenance and reliever treatment. However, blood tests showed mild leukocytosis and elevated levels of acute-phase proteins. Allergy, stress, injury, surgery, or thyroid problems were all excluded. Complete blood tests repeated in our clinic confirmed neutrophilic leukocytosis (leukocyte count 15.59 × 10^3^ cell/mmc, normal value <11 × 10^3^ cell/mmc; neutrophil 10.84 × 10^3^ cell/mmc, normal value <7.2 × 10^3^ cell/mmc) and elevated levels of C-reactive protein (CRP) (3.46 mg/dl, cutoff value < 0.50) and erythrocyte sedimentation rate (ESR) (81 mm/h, cutoff value < 28). No other relevant findings emerged (normal liver and kidney function, electrophoresis, and urine analysis). Eosinophils were normal. ANCA testing proved negative. Free light chains were present at normal ranges in the blood. After hematological consult, lympho-proliferative disease was excluded and laboratory alterations were ascribed to a reactive leukocytosis derived from rheumatological condition.

**Figure 1:**
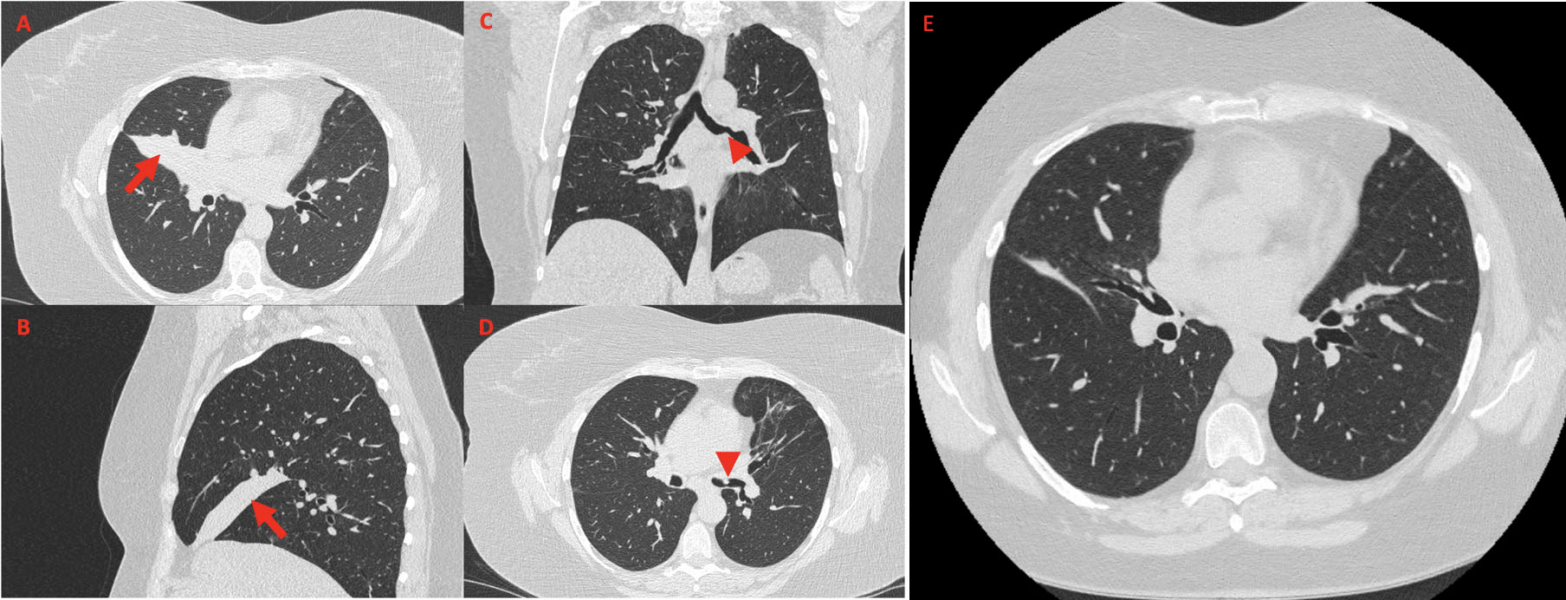
High-resolution chest CT showing a wedge-shaped density extending from the hilum anteriorly and inferiorly towards the chest wall, confirming the presence of middle lobe syndrome [red arrow in **(A)**, axial view, and **(B)**, sagittal view]. Coronal view **(C)** shows polypoid-like neoformation of the anterior wall of the left common bronchus (red arrowhead), confirmed in axial view (**D**, red arrowhead). High-resolution chest CT performed 15 days after the bronchodilation procedure documented regression of the middle lobe atelectasis (**E**, axial view).

Because of persistent leukocytosis and elevated inflammatory markers, in the absence of alternative causes, and as the patient's last chest CT scan dated 2 years before, in agreement with the pulmonary specialist, an HRCT examination (**Figures 1A–D**) was scheduled again, highlighting complete bronchiectasis and atelectasis of the middle lobe (ML), on an obstructive basis because of stenosis at the origin of the ML bronchial branch (**Figures 1A, B**). Moreover, diffuse thickening of the proximal bronchial walls of the inferior ipsilateral lobar branch, the common bronchus of the left lobe, and the origin of the bronchial branches of the lingula were detected. A polypoid-like neoformation of the anterior wall of the left common bronchus was reported as well, measuring approximately 8 × 10 mm (**Figures 1C, D**). According to a radiologist and pneumologist consultant, these findings appeared worthy of endoscopic investigations to rule out differential diagnosis between granulomas and heterotoplastic tissue.

**Figure 2:**
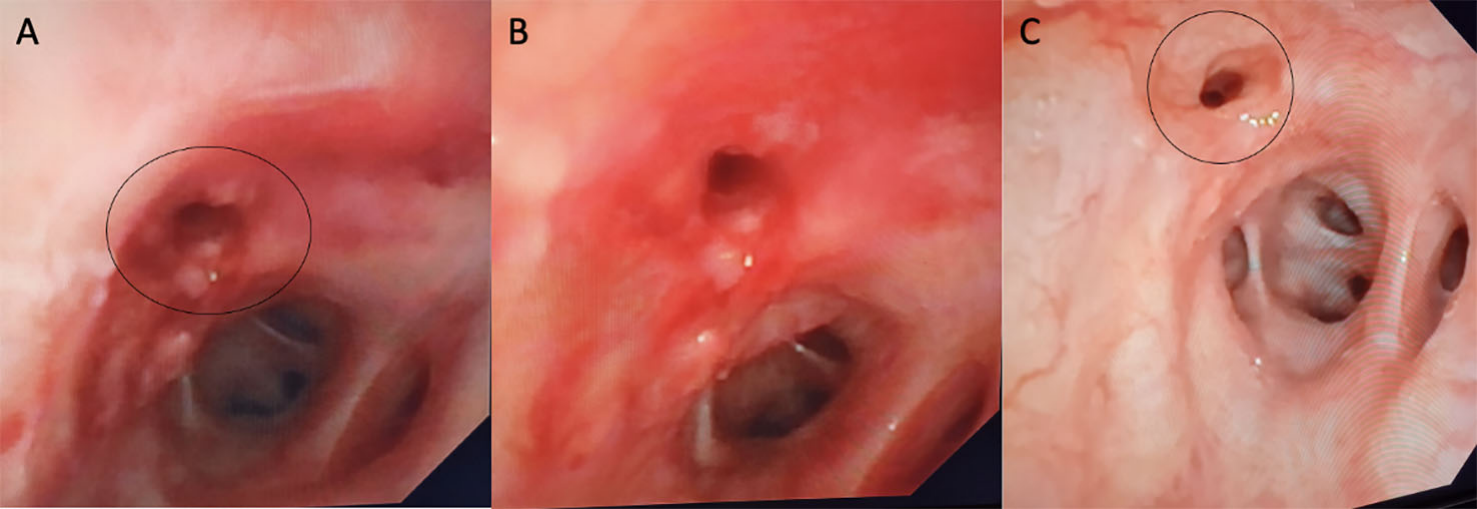
On bronchoscopy, the right main bronchus was partially obstructed by fibrotic stenosis **(A)**. **(B)** shows bronchoscopic images after dilatation and 1 month later showing complete healing of bronchial tear **(C)**.

After fiberoptic bronchoscopy (FOB), stenosis at the origin of the middle lobar bronchus, associated with architectural stenosis of the distal section of the left main bronchus, was confirmed (**Figure 2A**). At BAL, no hypereosinophilia was detected and microbiological tests were all negative for infections.

**Figure 3:**
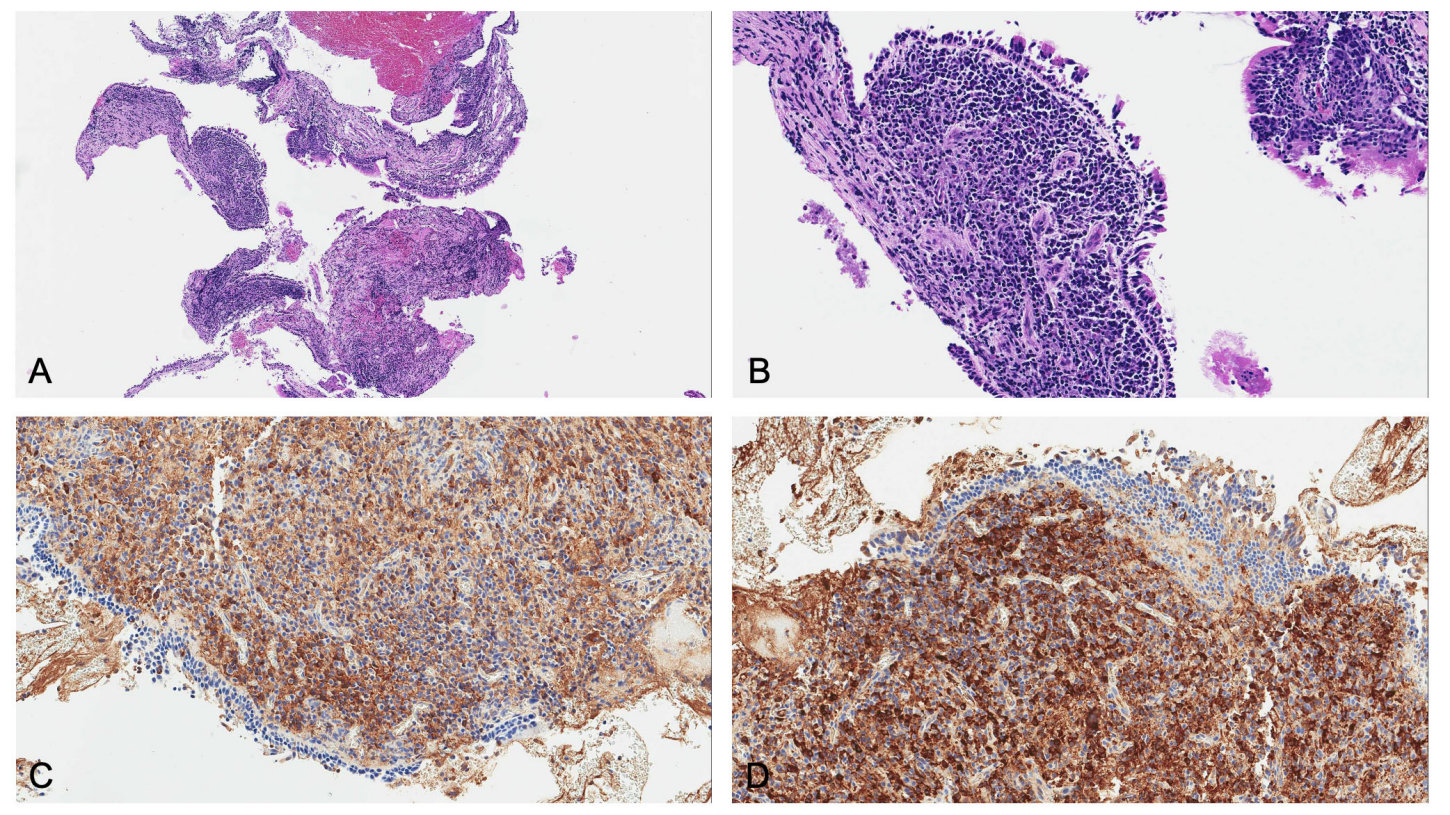
Microscopic examination revealed: **(A)** low magnification and **(B)** high magnification of hematoxylin and eosin staining, showing plasma cell infiltrate in the absence of eosinophils and vasculitis. Since main differential diagnosis includes lymphoma, kappa and lambda light chain immunostains were needed. Immunoglobulin free light chain (IgLC) levels in lung tissue were determined by immunohistochemistry, and no abnormalities were observed both at **(C)** low and **(D)** high magnification.

Bronchial biopsies showed the prevalence of an infiltrate of plasma cells and neutrophils, with isolated eosinophils, in the absence of granuloma, fibrinoid necrosis, eosinophilic vasculitis, and neoplastic cells (**Figure 3**). Nonabundant interstitial IgG4 plasma cells by IgG4 immunoperoxidase stain were observed, the Tfh–plasmablast axis was not elevated, and no positive stains to CD138, CD38, MUM1, and CD79a were observed. Based on these features, the pneumologist concluded an MLS diagnosis. Anti-inflammatory therapy was attempted, with prednisone 25 mg daily for 35 days. Because of lack of response to conservative treatment, endoscopic attempt of middle lobar bronchus dilatation (by Fogarty 4 F balloon and tissue removal using flexible biopsy forceps) was scheduled (**Figure 2B**). At follow-up chest CT scan, reventilation of middle lobe was documented (**Figure 1E**). Repeated FOB confirmed improving middle lobar bronchus stenosis (**Figure 2C**). The patient completely recovered from the procedure, without any respiratory symptom. Complete blood count was repeated 20 days after the procedure, documenting a slowly lowering neutrophilic leukocytosis (leukocyte count 14.3 × 10^3^ cell/mmc, normal value <11 × 10^3^ cell/mmc; neutrophil 9.9 × 10^3^ cell/mmc, normal value <7.2 × 10^3^ cell/mmc), as well as CRP (1.71 mg/dl, cutoff value < 0.50) and ESR (72 mm/h, cutoff value < 28).

## Discussion

Lung involvement is one of the most common clinical features in AAV (9). Airway involvement presents as tracheobronchial (TB) obstruction, sustained by inflammation, which occurs at any region of the tracheobronchial tree, including subglottic stenosis (SGS) and lower tracheal and bronchial stenosis (BS). Notably, BS is less frequently described, since it is more frequently asymptomatic and discovered accidentally, unlike SGS, which becomes rapidly symptomatic (voice changes, noisy breathing, dyspnea, etc.). Thus, BS is probably underdiagnosed among AAV patients (10). TB involvement is more frequent in GPA (15%–55% of patients, according to different cohorts in literature) (10–12), but it is rarely described even in MPA and EGPA (13).

MLS is a rare clinical entity, defined as chronic or recurrent atelectasis of the right middle lobe of the lung, and it is a potential complication of asthma. In some cases, the lingula of the left lung may also be involved (6).

To the best of our knowledge, this is the first detailed description of MLS in EGPA. Although there is no established definition of MLS, it is fundamentally distinct into two pathophysiological pathways: a non-obstructive type (patent right middle lobe bronchus) and an obstructive type (documented airway occlusion), both caused by various etiologies. A history of atopy, asthma, or COPD has been reported in up to 50% of patients (14).

Recurrent infections and chronic airway inflammatory processes, as observed in asthma, may lead to parenchymal damage and bronchiectasis, and contribute to transient bronchial obstruction (15).

TB obstruction/stenosis differential diagnosis is thus mandatory. Infections, traumatic injuries, radiation, and extrinsic compression (e.g., lung malignancies) are the principal etiologies. Other rarer causes are rheumatoid arthritis (RA), sarcoidosis, amyloidosis, Behçet's disease, relapsing polychondritis (RP), and fibrosing mediastinitis due to autoimmune disorders such as IgG4-RD (16–25).

Nonetheless, histological sampling of tracheobronchial lesions is not always specific (10). It often shows necrotizing granulomas that alter the normal alveolar architecture, progressively impairing respiratory function and causing subsequent bronchial stenosis and fibrosis. However, also non-specific mucosal inflammation and ulcerations may be observed, and it has to be considered that vasculitis is much more rarely encountered compared to granuloma, such as in our case (26).

It is important that the bronchoscopist carefully inspects the airways, since the TB mucosal lesions may be easily missed as they are very tiny and possibly sparse (27).

Since BS should be considered a severe manifestation of AAV, eventually leading to marked functional and life-threatening risks, physicians should be aware of this complication.

It may present totally asymptomatic, showing solely raised leukocyte count and inflammatory markers, in the absence of other possible explanation, similarly to our reported case (9).

Commonly, asthma exacerbation should be ruled out, and BS diagnosis may therefore derive only from chest HRCT, scheduled to exclude the presence of asthma abnormalities, namely, air trapping, bronchiectasis, bronchial dilatation, and bronchial wall thickening (28–31). In BS, imaging reports chronic or recurrent lobe atelectasis, particularly involving ML, since ML’s fissures insufflate the segment from collateral ventilation, reducing the likelihood of auto-correction of atelectasis (32), configuring MLS disease.

Findings from literature data suggest that BS involvement might be significantly more frequent than generally believed in patients being evaluated for possible eosinophilic lung diseases. In fact, chronic inflammation, as observed in recurrent asthma episodes and eosinophilic infiltrates, may lead to altered bronchus mucosal microarchitecture and fibrosis, thus causing BS (6). Therefore, careful asthma management in these patients is decisive to prevent airway stenoses and lobar collapse (33).

**Figure 4:**
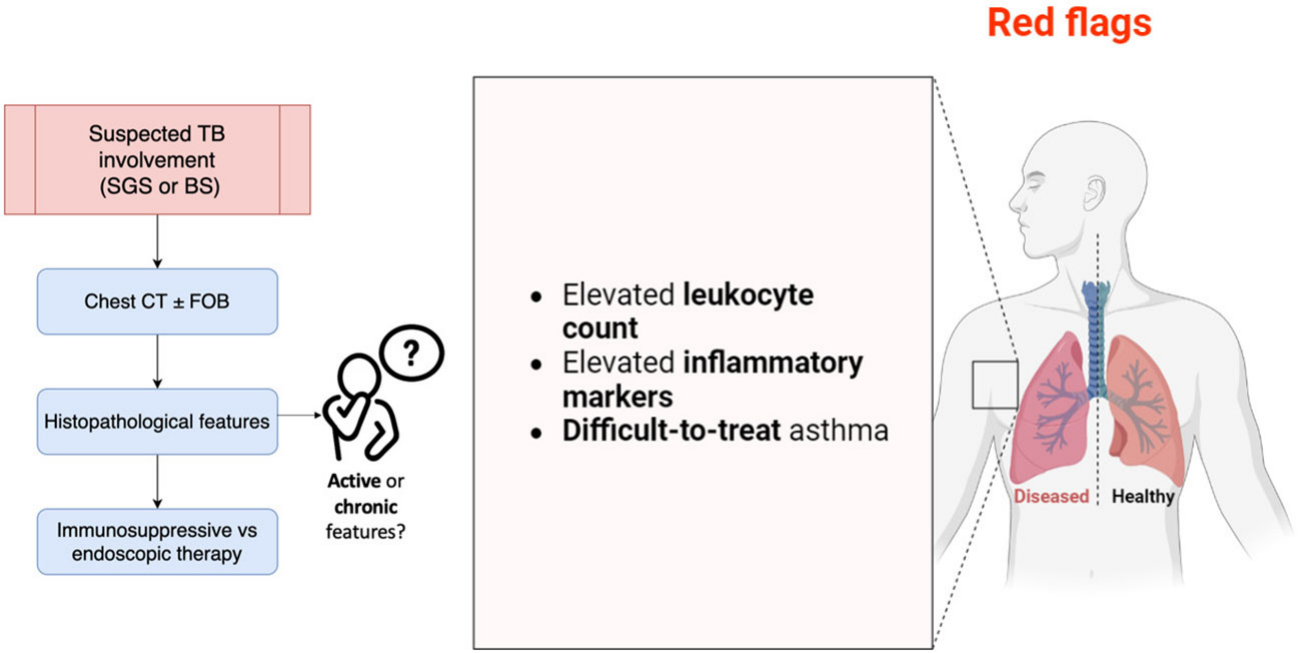
Main points to consider in AAV patients at risk of MLS development. BS, bronchial stenosis; CT, computed tomography; FOB, fiberoptic bronchoscopy; SGS, subglottic stenosis; TB, tracheobronchial. The image was created with BioRender.

**Figure 4** summarizes the main aspects discussed and to be considered in AAV patients at risk of MLS development.

Regarding management, TB disease may require treatment with high-dose systemic glucocorticoids and cyclophosphamide or rituximab. In a small cohort in literature, cyclophosphamide seemed to effectively treat BS, but not SGS, while rituximab appeared to be a promising therapy for tracheobronchial lesions (11, 34).

When TB involvement does not respond to standard immunosuppressive treatment, particularly when the stenosis is severe or it is likely to be problematic in the future (e.g., potentially complicating intubation or decannulation), and especially in symptomatic patients, complementary endoscopic management may be considered (35).

Furthermore, TB disease evolves independently from other systemic AAV manifestations, and little is known in terms of therapeutic response or evolution, even if viral infection and autoimmune flares may play a role (36). ANCA autoantibodies and inflammatory markers could be useful in monitoring disease course (37); however, they may be non-specific, affected by other causes (e.g., infections, as above mentioned), and they fluctuate over time without effectively predicting disease flares (38). Moreover, TB stenosis relapses are common and mostly occur under immunosuppressant therapy, suggesting the absence of response to classical AAV treatment (10). Differentiating active inflammatory endobronchial involvement from damage (scarring resulting from post-inflammatory fibrosis, or restenosis) may be clinically challenging, especially in patients without any other active organ involvement or in patients with negative ANCA test results, like in our presented case. In such patients, enforcing an intensification of immunosuppressive therapy may prove ineffective, merely increasing the risk of infectious complications (3). The CRP trend in our patient was decreasing after operative bronchus dilatation, and we believe this may reflect chronic BS. Otherwise, ongoing inflammation and fibroblast activation may subtend active disease. Additionally, characterization of serial changes in inflammation in EGPA patients with lung involvement may provide information about disease progression, foster further imaging investigation, potentially allowing risk stratification, and finally helping clinicians through treatment plans. Managing other sources of inflammation that could accelerate or induce recurrence may be pivotal as well, even if, today, there are no guidelines supporting physicians in such cases, and the risk of recurrence remains undisclosed. In conclusion, the rationale for TB stenosis screening in AAV patients is based on clinical experience, since usually patients are completely asymptomatic. Laboratory markers are not helpful as disease biomarkers, but they could be considered “red flags”, identifying patients needed to be closely monitored. Prompt evaluation with chest CT and FOB is required, even if histopathology may be inconclusive in defining acute rather than chronic disease features. Immunosuppressive therapy is the gold standard, but if worsening stenosis is confirmed, endoscopic restoration of airway patency is indicated (39).

To the best of our knowledge, this is the first detailed description of MLS in EGPA completely removed through FOB. Identification of MLS in EGPA appears imperative as prognosis, longitudinal management, and treatment options may differ from other pulmonary involvement in AAV patients.

## Data availability statement

The original contributions presented in the study are included in the article/supplementary material. Further inquiries can be directed to the corresponding author.

## Ethics statement

Ethical review and approval was not required for the study on human participants in accordance with the local legislation and institutional requirements. The patients/participants provided their written informed consent to participate in this study. Written informed consent was obtained from the participant/patient(s) for the publication of this case report.

## Author contributions

Conceptualization, BM; methodology, BM, IG, GLC, MB, ALM, MG; investigation, BM, IG, GLC, MB, ALM; data curation, BM, IG, GLC, MB, ALM, MG; writing—original draft preparation, BM; writing—review and editing, BM, IG, GLC, MB, ALM, MG; supervision, MG. All authors contributed to the article and approved the submitted version.
